# Patient-Reported Outcomes After Septoplasty With Bilateral Radiofrequency Inferior Turbinate Reduction: A Prospective Cohort Study

**DOI:** 10.7759/cureus.116498

**Published:** 2026-09-18

**Authors:** Maria C Michali, Ioannis G Kastanioudakis, Ioannis D Komnos, Lentiona Basiari, Polyniki Kastanioudaki, Vasileios Danielidis, Alexandros Georgolios, Nafsika Ziavra

**Affiliations:** 1 Otorhinolaryngology, Head and Neck Surgery, University Hospital of Ioannina, Ioannina, GRC; 2 Otorhinolaryngolohy, School of Medicine, University of Patras, Patras, GRC; 3 Speech and Language Therapy, University of Ioannina, Ioannina, GRC

**Keywords:** nasal obstruction, nasal septum deviation, nose questionnaire, septoplasty, snot-22 questionnaire

## Abstract

Introduction: Nasal septal deviation (NSD) is a common anatomical condition that contributes to nasal obstruction and may significantly impair patients' quality of life. Septoplasty is a standard surgical treatment for symptomatic septal deviation and may be combined with inferior turbinate reduction when turbinate hypertrophy contributes to nasal obstruction. However, postoperative outcomes vary among patients, and the degree of symptom improvement is not always consistent. The aim of this study was to evaluate patient-reported outcomes following septoplasty with bilateral radiofrequency reduction of the inferior turbinates using the Nasal Obstruction Symptom Evaluation (NOSE) scale and the 22-item Sino-Nasal Outcome Test (SNOT-22) and to compare postoperative outcomes with those of healthy controls.

Methods: A total of 43 patients with symptomatic nasal septal deviation and nasal obstruction were prospectively enrolled, along with 20 healthy controls. All patients underwent conventional septoplasty with concomitant bilateral radiofrequency reduction of the inferior turbinates. Patients completed the validated Greek versions of the NOSE and SNOT-22 questionnaires before surgery and six months postoperatively. Healthy controls completed both questionnaires at baseline. Baseline demographic characteristics, including age, sex, and body mass index (BMI), were also recorded.

Results: Both NOSE and SNOT-22 scores improved significantly six months after the combined surgical intervention compared with preoperative values (p < 0.001). The mean improvement in SNOT-22 score was 56.51 ± 12.67 points, while the mean improvement in NOSE score was 57.44 ± 12.55 points. At six months, postoperative NOSE and SNOT-22 scores were not significantly different from those observed in healthy controls. Age and BMI were not significantly associated with preoperative NOSE or SNOT-22 scores, whereas male patients had significantly higher preoperative SNOT-22 scores than female patients.

Conclusions: Septoplasty with bilateral radiofrequency reduction of the inferior turbinates was associated with significant improvement in patient-reported nasal obstruction symptoms and sinonasal quality of life at six months, as reflected by marked reductions in NOSE and SNOT-22 scores. Postoperative questionnaire scores were not significantly different from those of healthy controls. Because both procedures were performed concomitantly in all patients, the relative contribution of septoplasty and inferior turbinate reduction to the observed improvement cannot be determined separately.

## Introduction

Nasal obstruction is among the most frequent symptoms encountered in rhinologic practice and represents a major cause of impaired health-related quality of life. Nasal septal deviation (NSD) is one of the principal anatomical causes, resulting in disruption of normal nasal airflow and leading to partial or complete obstruction of one or both nasal cavities. In addition to its anatomical characteristics, NSD has important functional consequences and may significantly impair patients' quality of life [1].

Septoplasty remains the standard surgical treatment for symptomatic septal deviation. Although it is widely performed and generally considered effective, reported outcomes vary, and some patients continue to experience persistent symptoms or dissatisfaction despite technically successful surgery. This suggests that anatomical correction alone may not fully explain persistent symptoms. Factors such as nasal valve dysfunction, allergic inflammation, mucosal disease, and lateral wall instability may contribute to persistent symptoms and influence postoperative functional outcomes [2,3].

Patient-reported outcome measures (PROMs) are increasingly used to evaluate the effectiveness of septoplasty. Among the most commonly used instruments are the Nasal Obstruction Symptom Evaluation (NOSE) questionnaire, which specifically assesses the severity of nasal obstruction, and the 22-item Sino-Nasal Outcome Test (SNOT-22) questionnaire, which evaluates a broader range of sinonasal symptoms and their impact on quality of life [4-6]. The NOSE scale is a disease-specific, highly responsive instrument designed to assess the functional severity of nasal obstruction and is widely used in studies evaluating septoplasty outcomes [5,7]. Although the SNOT-22 was originally developed for chronic rhinosinusitis, it has also been shown to correlate well with NOSE scores and is increasingly used to assess outcomes in patients with nasal obstruction [8].

Previous studies have generally reported improvement in symptoms and quality of life after septoplasty, although the magnitude and durability of benefit vary across patient cohorts. A systematic review of long-term patient-reported outcomes found improvement in nasal obstruction after surgery but also highlighted considerable heterogeneity in reported outcomes and patient satisfaction [3]. In a study comparing patients undergoing septoplasty with healthy individuals, Bugten et al. reported substantial postoperative improvement, although some symptoms and quality-of-life measures remained worse than those observed in the control group [4]. Earlier prospective work also demonstrated improvement in nasal symptoms and general health following septal surgery [9]. Efforts to identify predictors of surgical outcome have produced less consistent findings; Cantone et al. reported that characteristics of the septal deviation were more closely associated with postoperative functional improvement than demographic variables [10]. These findings support the use of validated patient-reported outcome measures and provide further rationale for comparing postoperative results with those of healthy individuals.

Despite the widespread use of these instruments, it remains unclear to what extent patient-reported outcomes after surgical treatment for symptomatic NSD approach those of healthy individuals. In addition, the influence of demographic and anthropometric factors on subjective symptom burden has not been fully established. Therefore, the present prospective study evaluated patient-reported outcomes following conventional septoplasty with concomitant bilateral radiofrequency reduction of the inferior turbinates using validated PROMs and compared postoperative results with those of healthy controls [9,10].

## Materials and methods

Study design and participants

A prospective observational study was conducted at the Department of Otorhinolaryngology of the University Hospital of Ioannina, Greece, between January 2023 and January 2024. The study included patients with symptomatic NSD who underwent conventional septoplasty with concomitant bilateral radiofrequency reduction of the inferior turbinates for persistent nasal obstruction. Ethical approval was obtained from the Scientific Review Board of the University General Hospital of Ioannina (Approval No. 2/28-1-2021) before study initiation.

The primary objective was to evaluate changes in patient-reported nasal obstruction and sinonasal quality of life following conventional septoplasty with concomitant bilateral radiofrequency reduction of the inferior turbinates using the validated Greek versions of the NOSE scale and the SNOT-22 [11,12]. Secondary analyses examined the association of age, sex, and body mass index (BMI) with preoperative symptom severity.

The surgical patient group consisted of individuals undergoing functional nasal surgery for symptomatic nasal obstruction. No formal a priori sample-size or power calculation was performed. The sample size was determined by the number of eligible patients who agreed to participate during the predefined recruitment period. A total of 63 patients were assessed for eligibility. Of these, 11 did not meet the eligibility criteria and nine declined participation. The remaining 43 eligible patients who agreed to participate were consecutively enrolled in the study. The inclusion and exclusion criteria for patient enrollment are summarized in Table 1. A previous history of nasal trauma was not considered an exclusion criterion. Patients with prior nasal trauma were eligible for inclusion provided that they met the remaining eligibility criteria. 

**Table 1:** Inclusion and exclusion criteria for patient enrollment

Inclusion criteria	Exclusion criteria
Age 18–65 years	Age <18 or >65 years
Symptomatic nasal obstruction secondary to nasal septal deviation	Previous nasal surgery
Failure of at least 3 months of conservative medical treatment	Chronic rhinosinusitis
Scheduled to undergo septoplasty with bilateral radiofrequency inferior turbinate reduction	Nasal valve dysfunction confirmed by a positive Cottle maneuver
Completed preoperative and 6-month postoperative evaluations	Asthma
Provided written informed consent	Autoimmune disease
	Diabetes mellitus
	Cardiovascular disease
	History of head and neck radiotherapy
	Sinonasal malignancy

The control group consisted of 20 healthy volunteers between 18 and 65 years of age without chronic nasal obstruction, previous sinonasal surgery, or clinically significant sinonasal disease. Control participants completed the NOSE and SNOT-22 questionnaires once at the time of recruitment.

Medical history, demographic characteristics, and anthropometric measurements were recorded for all participants. All participants underwent clinical examination, including nasal endoscopy. Endoscopic findings were not prospectively recorded as prespecified quantitative study variables and were not assessed using a standardized endoscopic scoring system; therefore, they were not included in the statistical analysis. Patients completed the NOSE and SNOT-22 questionnaires before surgery and six months after surgery, whereas healthy controls completed both questionnaires once at baseline.

Surgical technique

All patients underwent conventional septoplasty using the Cottle technique through a hemitransfixion incision. The procedure was performed under direct visualization and was not endoscopically assisted. Deviated cartilaginous and/or bony septal components were corrected as required to achieve adequate septal alignment while preserving structural support. Bilateral radiofrequency reduction of the inferior turbinates was performed concomitantly in all patients; no formal turbinoplasty was performed. All procedures were performed by the same surgical team to ensure consistency in the operative technique.

Outcome measures

The NOSE questionnaire consists of five items evaluating nasal congestion, nasal obstruction, difficulty breathing through the nose, sleep disturbance, and difficulty obtaining sufficient airflow during physical exertion. Each item is scored from 0 (not a problem) to 4 (severe problem). The total raw score ranges from 0 to 20 and is multiplied by five, resulting in a final score ranging from 0 to 100.

The SNOT-22 questionnaire consists of 22 items evaluating sinonasal symptoms and their impact on quality of life. Each item is scored from 0 (no problem) to 5 (problem as bad as it can be), resulting in a total score ranging from 0 to 110. Only total questionnaire scores were included in the statistical analysis.

The validated Greek versions of both questionnaires were used. The NOSE scale was used in accordance with its published terms of use, with appropriate citation of the original validation study. Written permission was obtained for the use of the SNOT-22 in the present academic study and for publication of the resulting data.

Ethical considerations

The study was conducted in accordance with the ethical principles of the Declaration of Helsinki. Written informed consent was obtained from all participants before enrollment. Participant confidentiality and anonymity were maintained throughout the study.

Statistical analysis

Statistical analysis was performed using IBM SPSS Statistics for Windows, Version 27 (Released 2020; IBM Corp., Armonk, New York). Continuous variables were expressed as mean ± standard deviation (SD), whereas categorical variables were presented as frequencies and percentages. Normality of continuous variables was assessed using the Shapiro-Wilk test before parametric analyses were performed. Correlations between age, BMI, and preoperative NOSE and SNOT-22 scores were assessed using Pearson's correlation coefficient. Comparisons between preoperative and postoperative questionnaire scores were performed using paired-samples t-tests. Postoperative questionnaire scores of surgically treated patients were compared with those of healthy controls using independent-samples t-tests. Differences in preoperative questionnaire scores between male and female patients were evaluated using independent-samples t-tests. A two-sided p-value < 0.05 was considered statistically significant.

## Results

Baseline characteristics

The final study sample comprised 43 surgical patients and 20 healthy controls (63 participants in total). The baseline demographic characteristics of the study population are presented in Table 2. The mean age of the patient group was 35.21 ± 11.64 years, compared with 34.55 ± 9.96 years in the healthy control group. The corresponding mean body mass index (BMI) was 23.56 ± 3.10 kg/m² and 25.07 ± 3.88 kg/m², respectively. Males accounted for 65.1% (28/43) of patients in the surgical group and 65.0% (13/20) of healthy controls, while females represented 34.9% (15/43) and 35.0% (7/20) of the two groups, respectively. The baseline demographic characteristics were comparable between the two groups, with no significant differences in age, BMI, or sex distribution. 

**Table 2:** Baseline demographic characteristics of the study population Data are presented as mean ± SD or n (%). BMI: body mass index; SD: standard deviation.

Variable	Patients (n = 43)	Healthy controls (n = 20)
Age (years), mean ± SD	35.21 ± 11.64	34.55 ± 9.96
BMI (kg/m²), mean ± SD	23.56 ± 3.10	25.07 ± 3.88
Male sex, n (%)	28 (65.1)	13 (65.0)
Female sex, n (%)	15 (34.9)	7 (35.0)

Descriptive analysis of questionnaire scores

Descriptive statistics for the NOSE and SNOT-22 questionnaires are presented in Table 3. Patients reported high preoperative symptom scores on both questionnaires. The mean preoperative NOSE score was 86.63 ± 10.16 (median, 85; range, 65-100), decreasing to 29.19 ± 6.72 (median, 30; range, 10-40) six months after the combined surgical intervention. The corresponding mean NOSE score in the healthy control group was 27.00 ± 2.20 (median, 26; range, 23-30). Similarly, the mean preoperative SNOT-22 score was 84.63 ± 13.51 (median, 85; range, 58-105), decreasing to 28.12 ± 5.18 (median, 27; range, 19-41) postoperatively. Healthy controls had a mean SNOT-22 score of 27.65 ± 7.18 (median, 29; range, 14-42). Overall, descriptive statistics demonstrated markedly lower postoperative NOSE and SNOT-22 scores compared with preoperative values.

**Table 3:** Descriptive statistics of NOSE and SNOT-22 questionnaire scores Data are presented as mean ± SD, median, and range. NOSE: Nasal Obstruction Symptom Evaluation; SNOT-22: 22-item Sino-Nasal Outcome Test; SD: standard deviation.

Variable	Mean ± SD	Median	Range
Preoperative NOSE	86.63 ± 10.16	85	65–100
Postoperative NOSE	29.19 ± 6.72	30	10–40
Healthy controls NOSE	27.00 ± 2.20	26	23–30
Preoperative SNOT-22	84.63 ± 13.51	85	58–105
Postoperative SNOT-22	28.12 ± 5.18	27	19–41
Healthy controls SNOT-22	27.65 ± 7.18	29	14–42

NOSE score improvement

Comparison of preoperative and postoperative NOSE scores is presented in Table 4. A statistically significant reduction in NOSE scores was observed six months after the combined surgical intervention (t = 30.006, df = 42, p < 0.001). The mean improvement was 57.44 ± 12.55 points, with a 95% confidence interval of 53.58 to 61.31 points.

**Table 4:** Comparison of preoperative and postoperative NOSE scores Data are presented as mean difference ± SD with 95% confidence intervals (CI). SD: standard deviation; CI: confidence interval; df: degrees of freedom; NOSE: Nasal Obstruction Symptom Evaluation.

Variable	Mean difference ± SD	95% CI	t	df	P
Preoperative NOSE score − Postoperative NOSE score	57.44 ± 12.55	53.58 to 61.31	30.006	42	<0.001

SNOT-22 score improvement

Comparison of preoperative and postoperative SNOT-22 scores is presented in Table 5. A statistically significant reduction in SNOT-22 scores was observed six months after the combined surgical intervention (t = 29.260, df = 42, p < 0.001). The mean improvement was 56.51 ± 12.67 points, with a 95% confidence interval ranging from 52.61 to 60.41 points.

**Table 5:** Comparison of preoperative and postoperative SNOT-22 scores Data are presented as mean difference ± SD with 95% confidence intervals (CI). SD: standard deviation; CI: confidence interval; df: degrees of freedom; SNOT-22: 22-item Sino-Nasal Outcome Test.

Variable	Mean difference ± SD	95% CI	t	df	p
Preoperative SNOT-22 score − Postoperative SNOT-22 score	56.51 ± 12.67	52.61 to 60.41	29.260	42	<0.001

Comparison between postoperative patients and healthy controls

Postoperative NOSE and SNOT-22 scores were compared between the surgical patient group and healthy controls. The results are summarized in Table 6. The mean postoperative NOSE score was 29.19 ± 6.72 in the surgical patient group and 27.00 ± 2.20 in the healthy control group. No statistically significant difference was observed between the two groups (independent-samples t-test, equal variances assumed: t = -1.415, df = 61, p = 0.162). The mean difference was -2.19 points (95% CI: -5.28 to 0.90). Similarly, the mean postoperative SNOT-22 score was 28.12 ± 5.18 in the patient group and 27.65 ± 7.18 in the healthy control group. The difference between groups was also not statistically significant (independent-samples t-test, equal variances assumed: t = -0.293, df = 61, p = 0.770), with a mean difference of -0.47 points (95% CI: -3.65 to 2.71). For both questionnaires, the 95% confidence intervals included zero, indicating no statistically significant differences between postoperative patients and healthy controls.

**Table 6:** Comparison of postoperative NOSE and SNOT-22 scores between surgical patients and healthy controls Values are presented as mean ± SD. NOSE: Nasal Obstruction Symptom Evaluation; SNOT-22: 22-item Sino-Nasal Outcome Test; SD: standard deviation; CI: confidence interval; df: degrees of freedom. Mean difference was calculated as healthy controls minus postoperative surgical patients.

Questionnaire	Group	n	Mean ± SD	Mean difference (95% CI)	t	df	p-value
NOSE	Healthy controls	20	27.00 ± 2.20	-2.19 (-5.28 to 0.90)	-1.415	61	0.162
	Postoperative surgical patients	43	29.19 ± 6.72	—	—	—	—
SNOT-22	Healthy controls	20	27.65 ± 7.18	-0.47 (-3.65 to 2.71)	-0.293	61	0.770
	Postoperative surgical patients	43	28.12 ± 5.18	—	—	—	—

As shown in Table 7, no statistically significant correlation was observed between age and preoperative NOSE scores (r = −0.085, p = 0.586). Similarly, no significant association was identified between age and preoperative SNOT-22 scores (r = −0.050, p = 0.748). These results indicate that age was not significantly associated with preoperative nasal obstruction severity or sinonasal quality-of-life impairment.

**Table 7:** Correlation between age and preoperative questionnaire scores Pearson correlation coefficients (r) and corresponding p-values are presented. NOSE: Nasal Obstruction Symptom Evaluation; SNOT-22: 22-item Sino-Nasal Outcome Test.

Variables	Correlation coefficient (r)	p-value
Age vs. preoperative NOSE	-0.085	0.586
Age vs. preoperative SNOT-22	-0.050	0.748

As shown in Table 8, no statistically significant correlation was observed between BMI and preoperative NOSE scores (r = 0.125, p = 0.426). Similarly, no significant association was identified between BMI and preoperative SNOT-22 scores (r = −0.044, p = 0.779). BMI was not significantly associated with preoperative nasal obstruction severity or sinonasal quality-of-life impairment.

**Table 8:** Correlation between BMI and preoperative questionnaire scores Pearson correlation coefficients (r) and corresponding p-values are presented. BMI: body mass index; NOSE: Nasal Obstruction Symptom Evaluation; SNOT-22: 22-item Sino-Nasal Outcome Test.

Variables	Correlation coefficient (r)	p-value
BMI vs. preoperative NOSE	0.125	0.426
BMI vs. preoperative SNOT-22	-0.044	0.779

As shown in Table 9, no statistically significant sex-related difference was observed in preoperative NOSE scores (p = 0.662). However, male patients had significantly higher preoperative SNOT-22 scores than female patients (87.93 vs. 78.47, p = 0.045). This difference suggests greater sinonasal quality-of-life impairment among male patients before surgery.

**Table 9:** Sex differences in preoperative questionnaire scores Comparison of preoperative questionnaire scores between male and female participants. Data are presented as mean ± SD. NOSE: Nasal Obstruction Symptom Evaluation; SNOT-22: 22-item Sino-Nasal Outcome Test.

Variable	Male mean ± SD	Female mean ± SD	p-value
Preoperative NOSE	87.14 ± 10.040	85.67 ± 10.668	0.662
Preoperative SNOT-22	87.93 ± 11.588	78.47 ± 15.028	0.045

Overall, no significant associations were identified between age or BMI and preoperative symptom severity, whereas sex was associated only with preoperative SNOT-22 scores. Age and BMI were not associated with preoperative symptom burden in this cohort, while higher SNOT-22 scores were observed among male patients.

## Discussion

Septoplasty is among the most commonly performed procedures in otorhinolaryngology and remains the standard surgical treatment for symptomatic nasal septal deviation refractory to medical therapy [4]. Despite its widespread use, postoperative outcomes may vary considerably, emphasizing the importance of evaluating surgical success not only anatomically but also through PROMs. In the present study, postoperative outcomes were assessed using the NOSE questionnaire and the SNOT-22, while postoperative results were also compared with those of healthy controls. In addition, the influence of demographic and anthropometric factors on preoperative symptom severity was investigated [13-16].

The principal findings were: (1) significant postoperative improvement in both NOSE and SNOT-22 scores, (2) postoperative NOSE and SNOT-22 scores that were not significantly different from those of healthy controls, (3) no significant associations between age or body mass index and preoperative symptom severity, and (4) a significant association between sex and preoperative SNOT-22 scores.

One of the main findings was the substantial reduction in NOSE scores six months after the combined surgical intervention. Patients demonstrated severe nasal obstruction before surgery, with a mean preoperative NOSE score of 86.63 ± 10.16, which decreased to 29.19 ± 6.72 postoperatively. This marked improvement reflects substantial symptomatic relief following surgical intervention. These findings are consistent with the original validation study by Stewart et al., who demonstrated that the NOSE questionnaire is highly responsive to surgical intervention and capable of detecting clinically meaningful postoperative changes [5]. Similarly, Rhee et al. identified the NOSE scale as one of the most reliable and responsive instruments for evaluating outcomes following functional nasal surgery [16]. Together, these findings support the effectiveness of surgical treatment for symptomatic nasal obstruction, although in the present cohort, the improvement reflects the combined effect of septoplasty and inferior turbinate radiofrequency reduction.

Substantial improvement was also observed in overall sinonasal quality of life, as reflected by the marked reduction in SNOT-22 scores. Preoperative SNOT-22 values indicated considerable impairment before surgery, whereas postoperative scores were numerically similar to those observed in healthy controls. Because the SNOT-22 questionnaire evaluates not only rhinologic symptoms but also sleep dysfunction, emotional well-being, fatigue, and social impairment, the observed postoperative reduction suggests broad improvement across multiple dimensions of patient health status. These findings are consistent with the psychometric validation study by Hopkins et al., who demonstrated the excellent responsiveness of SNOT-22 to changes in sinonasal disease severity [13]. Similar postoperative improvements have also been reported after septoplasty, with recent systematic review evidence demonstrating significant improvements in nasal obstruction symptoms and disease-specific quality of life following surgery [17].

The improvement observed in our cohort is consistent with previous reports of favorable patient-reported outcomes after septoplasty. In their systematic review of long-term outcomes, Tsang et al. found improvement in nasal obstruction after surgery, although the degree of symptom relief and patient satisfaction varied considerably across studies [3]. Earlier prospective work by Arunachalam et al. also demonstrated improvement in nasal symptoms and general health following septal surgery [9]. These findings support the overall effectiveness of septoplasty while also indicating that the magnitude of perceived benefit may vary according to patient selection, baseline symptom severity, associated nasal pathology, surgical management, and the outcome measure used.

A distinctive feature of the present study was the comparison of postoperative outcomes with those of healthy controls. Although postoperative NOSE scores remained slightly higher than those of the control group, the difference was not statistically significant, while postoperative SNOT-22 scores were also not significantly different from those observed in healthy individuals. This finding differs somewhat from that of Bugten et al., who reported significant improvement after septoplasty but found that postoperative quality-of-life scores remained worse than those of healthy controls [4]. The two studies are not directly comparable, however, because Bugten et al. used the SNOT-20 rather than the SNOT-22, and differences in patient selection and surgical management may also have influenced the findings. However, the absence of statistically significant differences should not be interpreted as evidence of equivalence between the surgical and control groups. Direct comparisons with healthy control populations remain relatively uncommon in the rhinologic literature, as most previous studies have focused on preoperative and postoperative comparisons within surgical cohorts. The inclusion of a healthy control group therefore provides additional clinical context for interpreting postoperative recovery.

No significant associations were identified between age or body mass index and preoperative NOSE or SNOT-22 scores, suggesting that these variables were not major determinants of subjective symptom burden in the present cohort. Previous studies have also suggested that demographic characteristics may have limited or variable effects on patient-reported nasal obstruction [16].

Male patients demonstrated significantly higher preoperative SNOT-22 scores than female patients, whereas no significant sex-related difference was observed for NOSE scores. Given the relatively small sample size and the absence of a prespecified analysis designed to investigate the reasons for this difference, this finding should be regarded as hypothesis-generating. The present data do not allow conclusions to be drawn regarding the mechanisms underlying this association, and confirmation in larger studies is required.

The present study has several strengths. Its prospective design and the use of two validated patient-reported outcome measures allowed changes in both nasal obstruction and broader sinonasal quality of life to be assessed. The inclusion of a healthy control group provided an additional reference for interpreting postoperative scores, while the use of the same operative approach by the same surgical team reduced variability related to surgical technique.

The study also has limitations. It was conducted at a single center and included a relatively small number of patients, which may limit the generalizability of the findings. No formal a priori sample-size or power calculation was performed. Follow-up was restricted to six months, and longer-term changes could therefore not be assessed. Objective measures of nasal airflow were not included in the present analysis, so postoperative improvement was evaluated on the basis of patient-reported outcomes. The healthy control group was smaller than the surgical group and completed the questionnaires only once at baseline; therefore, it served primarily as a clinical reference rather than as a longitudinal comparison group. Moreover, the study was not designed to establish equivalence between postoperative patients and healthy controls. In addition, all patients underwent bilateral radiofrequency reduction of the inferior turbinates at the time of septoplasty. The improvement observed after surgery therefore cannot be attributed to septoplasty alone, and the independent contribution of inferior turbinate treatment cannot be determined from this study.

From a clinical standpoint, the marked reductions in both NOSE and SNOT-22 scores indicate a meaningful symptomatic benefit following the combined surgical approach in patients with symptomatic nasal septal deviation and obstruction. At six months, postoperative NOSE and SNOT-22 scores were not significantly different from those recorded in the healthy control group, although this finding should not be interpreted as evidence of equivalence. The use of both NOSE and SNOT-22 also provided complementary information, capturing not only obstruction-specific symptoms but also the wider impact of sinonasal complaints on patients' daily well-being.

Future multicenter studies involving larger patient cohorts and longer follow-up are needed to further investigate postoperative outcomes in relation to healthy populations and to determine the long-term durability of these improvements.

## Conclusions

Septoplasty combined with bilateral radiofrequency reduction of the inferior turbinates was associated with substantial improvement in patient-reported nasal obstruction and sinonasal quality of life at six months. Both NOSE and SNOT-22 scores decreased markedly after surgery, and postoperative scores did not differ significantly from those of the healthy control group. These findings should not, however, be interpreted as evidence of equivalence between the two groups.

Age and body mass index were not related to preoperative symptom severity. Male patients had higher preoperative SNOT-22 scores than female patients, but this finding should be regarded as hypothesis-generating and requires confirmation in larger cohorts. Because all patients underwent both septoplasty and inferior turbinate radiofrequency reduction, the effect of each procedure cannot be assessed separately. The findings therefore reflect the outcome of the combined surgical approach and support the use of NOSE and SNOT-22 as complementary measures of postoperative patient-reported outcomes.
